# Triaqua­(7-oxabicyclo­[2.2.1]heptane-2,3-dicarboxyl­ato-κ^3^ *O* ^2^,*O* ^3^,*O* ^7^)cobalt(II) monohydrate

**DOI:** 10.1107/S1600536811028431

**Published:** 2011-07-23

**Authors:** Fan Zhang, Ai-Ping Jia, Qiu-Yue Lin

**Affiliations:** a Zhejiang Key Laboratory for Reactive Chemistry on Solid Surfaces, Institute of Physical Chemistry, Zhejiang Normal University, Jinhua, Zhejiang 321004, People’s Republic of China; b College of Chemistry and Life Science, Zhejiang Normal University, Jinhua 321004, Zhejiang, People’s Republic of China

## Abstract

The title complex, [Co(C_8_H_8_O_5_)(H_2_O)_3_]·H_2_O, was synthesized by reaction of cobalt acetate with 7-oxabicyclo­[2.2.1]heptane-2,3-dicarb­oxy­lic anhydride (norcantharidin) in aqueous solution. In the mol­ecule, the Co^II^ atom is six-coordinated in a distorted octa­hedral environment, binding to the bridging O atom of the bicyclo­heptane unit, to two O atoms from monodentate carboxyl­ate groups and to three water O atoms. The crystal structure is stabilized by several O—H⋯O hydrogen-bonding inter­actions involving both the coordinated and uncoordinated water mol­ecules as donors and the carboxyl­ate O atoms of neighbouring mol­ecules as acceptors.

## Related literature

For background to the applications of norcantharidin, see: Jiao *et al.* (2005 ▶); Wang (1989 ▶). For related structures, see: Wang *et al.* (2010 ▶); Kaplonek *et al.* (1994 ▶).
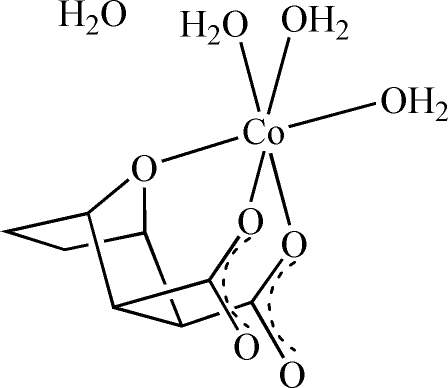


## Experimental

### 

#### Crystal data

[Co(C_8_H_8_O_5_)(H_2_O)_3_]·H_2_O
*M* *_r_* = 315.14
Monoclinic, 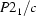

*a* = 10.0965 (3) Å
*b* = 10.0208 (3) Å
*c* = 14.5893 (3) Å
β = 129.177 (1)°
*V* = 1144.25 (5) Å^3^
*Z* = 4
Mo *K*α radiation
μ = 1.54 mm^−1^
*T* = 296 K
0.24 × 0.17 × 0.13 mm

#### Data collection

Bruker SMART CCD diffractometer
Absorption correction: multi-scan (*SADABS*; Sheldrick, 1996 ▶) *T*_min_ = 0.745, *T*_max_ = 0.824
14892 measured reflections
2004 independent reflections
1861 reflections with *I* > 2σ(*I*)
*R*_int_ = 0.021

#### Refinement

*R*[*F* ^2^ > 2σ(*F* ^2^)] = 0.023
*wR*(*F* ^2^) = 0.063
*S* = 1.08
2004 reflections
163 parameters
4 restraints
H-atom parameters constrained
Δρ_max_ = 0.28 e Å^−3^
Δρ_min_ = −0.30 e Å^−3^

### 

Data collection: *SMART* (Bruker, 2004 ▶); cell refinement: *SAINT* (Bruker, 2004 ▶); data reduction: *SAINT*; program(s) used to solve structure: *SHELXS97* (Sheldrick, 2008 ▶); program(s) used to refine structure: *SHELXL97* (Sheldrick, 2008 ▶); molecular graphics: *XP* in *SHELXTL* (Sheldrick, 2008 ▶); software used to prepare material for publication: *publCIF* (Westrip, 2010 ▶).

## Supplementary Material

Crystal structure: contains datablock(s) I, global. DOI: 10.1107/S1600536811028431/wm2510sup1.cif

Structure factors: contains datablock(s) I. DOI: 10.1107/S1600536811028431/wm2510Isup2.hkl

Additional supplementary materials:  crystallographic information; 3D view; checkCIF report

## Figures and Tables

**Table 1:** Selected bond lengths (Å)

Co1—O3	2.0631 (14)
Co1—O1*W*	2.0691 (15)
Co1—O2*W*	2.0728 (15)
Co1—O1	2.0849 (13)
Co1—O3*W*	2.0948 (13)
Co1—O5	2.1510 (13)

**Table 2:** Hydrogen-bond geometry (Å, °)

*D*—H⋯*A*	*D*—H	H⋯*A*	*D*⋯*A*	*D*—H⋯*A*
O2*W*—H2*WB*⋯O4*W*	0.85	2.06	2.872 (2)	160
O4*W*—H4*WB*⋯O5	0.85	2.60	3.0316 (19)	113
O1*W*—H1*WA*⋯O4^i^	0.85	1.88	2.716 (2)	169
O1*W*—H1*WB*⋯O4*W*^ii^	0.85	2.00	2.789 (2)	153
O2*W*—H2*WA*⋯O1^iii^	0.85	1.87	2.7168 (19)	171
O3*W*—H3*WB*⋯O2^iv^	0.85	1.84	2.688 (2)	173
O4*W*—H4*WB*⋯O2^iv^	0.85	2.09	2.916 (2)	164
O3*W*—H3*WA*⋯O3^v^	0.85	1.85	2.6969 (19)	178
O4*W*—H4*WA*⋯O3*W*^vi^	0.85	2.35	3.112 (2)	149

Symmetry codes: (i) 
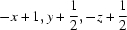
; (ii) 
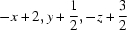
; (iii) 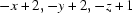; (iv) 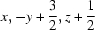; (v) 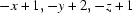; (vi) 
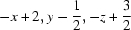
.

**Fig. 1.**
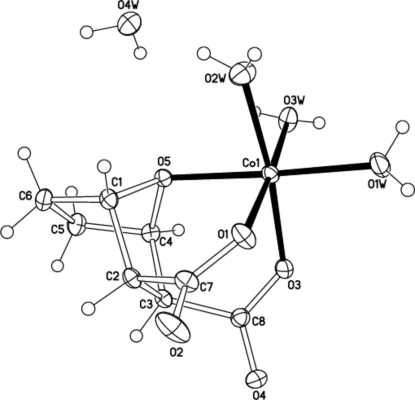
A view of the molecule of (I) showing the atom-labelling scheme with displacement ellipsoids drawn at the 30% probability level.

## supplementary crystallographic information

### Comment

7-oxabicyclo[2.2.1]heptane-2,3-dicarboxylic anhydride (norcantharidin), derived from cantharidin, is a variety of pharmacologically important compounds such as protein kinase inhibitors and antitumor properties (Wang, 1989). Cobalt is recognized as an essential metal element widely distributed in biological systems in cells and the body (Jiao *et al.*, 2005). A manganese complex of dimethylcantharate was reported recently (Wang *et al.*, 2010) and a similar cobalt complex of dimethylcantharate (Kaplonek *et al.*, 1994) has also been reported.

The molecular structure of the title complex is shown in Fig. 1. The cobalt(II) atom is six-coordinated in a distorted octahedral coordination mode, binding to the bridging O atom of the bicycloheptane unit, to two O atoms from corresponding carboxylate groups and to three O atoms from water. The crystal structure is stabilized by several O—H···O hydrogen-bonding interactions involving both the coordinated and uncoordinated water molecules as donors and the carboxylate O atoms of neighbouring molecules as acceptors.

### Experimental

An ethanol solution containing 0.5 mmol salicylic acid was dropwisely added into 0.5 mmol aqueous cobalt acetate solution. After stirring for one hour, an aqueous solution containing 0.5 mmol norcantharidin was dropwisely added into the mixture. Two hours later, the solution was filtered and after 2 weeks, crystals with suitable size for single-crystal X-ray diffraction were obtained.

### Refinement

H atoms bonded to C atoms were positioned geometrically and refined using a riding model [aliphatic of tertiary carbon C—H = 0.98 Å, aliphatic of secondary carbon C—H = 0.97 Å, both with *U*_iso_(H) = 1.2*U*_eq_(C)]. The H atoms bonded to O atoms were located in a difference Fourier maps and refined with O—H distance restraints of 0.85 (1) Å and *U*_iso_(H) = 1.5*U*_eq_(O).

### Crystal data

**Table:**

[Co(C_8_H_8_O_5_)(H_2_O)_3_]·H_2_O	*F*(000) = 652
*M**_r_* = 315.14	*D*_x_ = 1.829 Mg m^−^^3^
Monoclinic, *P*2_1_/*c*	Mo *K*α radiation, λ = 0.71073 Å
Hall symbol: -P 2ybc	Cell parameters from 8994 reflections
*a* = 10.0965 (3) Å	θ = 2.6–25.0°
*b* = 10.0208 (3) Å	µ = 1.54 mm^−^^1^
*c* = 14.5893 (3) Å	*T* = 296 K
β = 129.177 (1)°	Block, red
*V* = 1144.25 (5) Å^3^	0.24 × 0.17 × 0.13 mm
*Z* = 4	

### Data collection

**Table:**

Bruker SMART CCD diffractometer	2004 independent reflections
Radiation source: fine-focus sealed tube	1861 reflections with *I* > 2σ(*I*)
graphite	*R*_int_ = 0.021
ω scans	θ_max_ = 25.0°, θ_min_ = 2.6°
Absorption correction: multi-scan (*SADABS*; Sheldrick, 1996)	*h* = −12→11
*T*_min_ = 0.745, *T*_max_ = 0.824	*k* = −11→11
14892 measured reflections	*l* = −16→17

### Refinement

**Table:**

Refinement on *F*^2^	Primary atom site location: structure-invariant direct methods
Least-squares matrix: full	Secondary atom site location: difference Fourier map
*R*[*F*^2^ > 2σ(*F*^2^)] = 0.023	Hydrogen site location: inferred from neighbouring sites
*wR*(*F*^2^) = 0.063	H-atom parameters constrained
*S* = 1.08	*w* = 1/[σ^2^(*F*_o_^2^) + (0.0277*P*)^2^ + 1.0316*P*] where *P* = (*F*_o_^2^ + 2*F*_c_^2^)/3
2004 reflections	(Δ/σ)_max_ = 0.001
163 parameters	Δρ_max_ = 0.28 e Å^−^^3^
4 restraints	Δρ_min_ = −0.30 e Å^−^^3^

### Special details

**Table:**

Geometry. All e.s.d.'s (except the e.s.d. in the dihedral angle between two l.s. planes) are estimated using the full covariance matrix. The cell e.s.d.'s are taken into account individually in the estimation of e.s.d.'s in distances, angles and torsion angles; correlations between e.s.d.'s in cell parameters are only used when they are defined by crystal symmetry. An approximate (isotropic) treatment of cell e.s.d.'s is used for estimating e.s.d.'s involving l.s. planes.
Refinement. Refinement of *F*^2^ against ALL reflections. The weighted *R*-factor *wR* and goodness of fit *S* are based on *F*^2^, conventional *R*-factors *R* are based on *F*, with *F* set to zero for negative *F*^2^. The threshold expression of *F*^2^ > σ(*F*^2^) is used only for calculating *R*-factors(gt) *etc*. and is not relevant to the choice of reflections for refinement. *R*-factors based on *F*^2^ are statistically about twice as large as those based on *F*, and *R*- factors based on ALL data will be even larger.

### Fractional atomic coordinates and isotropic or equivalent isotropic displacement parameters (Å^2^)

**Table:**

	*x*	*y*	*z*	*U*_iso_*/*U*_eq_	
Co1	0.75127 (3)	0.93105 (2)	0.50125 (2)	0.01957 (10)	
O1	0.78435 (19)	0.89643 (14)	0.37592 (12)	0.0290 (3)	
O1W	0.7237 (2)	1.13465 (15)	0.47074 (14)	0.0427 (4)	
H1WA	0.7016	1.1781	0.4125	0.064*	
H1WB	0.7586	1.1869	0.5279	0.064*	
O2	0.7679 (2)	0.77531 (16)	0.24295 (14)	0.0442 (4)	
O2W	1.01188 (18)	0.94437 (15)	0.63877 (13)	0.0348 (4)	
H2WA	1.0847	0.9886	0.6399	0.052*	
H2WB	1.0612	0.8811	0.6887	0.052*	
O3	0.49581 (17)	0.89349 (14)	0.36612 (12)	0.0281 (3)	
O3W	0.71943 (19)	0.95345 (14)	0.62906 (13)	0.0301 (3)	
H3WA	0.6499	1.0012	0.6291	0.045*	
H3WB	0.7291	0.8840	0.6664	0.045*	
O4	0.3105 (2)	0.79264 (17)	0.19436 (13)	0.0465 (4)	
O4W	1.1014 (2)	0.73049 (16)	0.79981 (13)	0.0387 (4)	
H4WA	1.1328	0.6564	0.7907	0.058*	
H4WB	0.9987	0.7184	0.7718	0.058*	
O5	0.78301 (16)	0.72157 (12)	0.54243 (11)	0.0191 (3)	
C1	0.8565 (2)	0.64609 (19)	0.49869 (16)	0.0215 (4)	
H1A	0.9702	0.6772	0.5300	0.026*	
C2	0.7198 (2)	0.66254 (18)	0.36363 (16)	0.0206 (4)	
H2A	0.7197	0.5836	0.3239	0.025*	
C3	0.5510 (2)	0.66506 (18)	0.34899 (16)	0.0199 (4)	
H3A	0.4794	0.5884	0.3014	0.024*	
C4	0.6238 (2)	0.64553 (19)	0.47793 (16)	0.0209 (4)	
H4A	0.5468	0.6757	0.4930	0.025*	
C5	0.6883 (3)	0.5032 (2)	0.51937 (18)	0.0277 (4)	
H5A	0.6075	0.4384	0.4603	0.033*	
H5B	0.7105	0.4842	0.5932	0.033*	
C6	0.8554 (3)	0.5040 (2)	0.53581 (18)	0.0276 (4)	
H6A	0.9543	0.4871	0.6174	0.033*	
H6B	0.8519	0.4386	0.4853	0.033*	
C7	0.7572 (2)	0.7867 (2)	0.32282 (17)	0.0242 (4)	
C8	0.4448 (2)	0.79295 (19)	0.29760 (16)	0.0233 (4)	

### Atomic displacement parameters (Å^2^)

**Table:**

	*U*^11^	*U*^22^	*U*^33^	*U*^12^	*U*^13^	*U*^23^
Co1	0.02472 (16)	0.01767 (16)	0.01900 (16)	−0.00126 (9)	0.01509 (13)	−0.00144 (9)
O1	0.0461 (9)	0.0229 (7)	0.0333 (8)	−0.0087 (6)	0.0323 (7)	−0.0060 (6)
O1W	0.0740 (12)	0.0193 (8)	0.0271 (8)	−0.0025 (7)	0.0282 (8)	−0.0008 (6)
O2	0.0791 (12)	0.0378 (9)	0.0462 (10)	−0.0240 (8)	0.0541 (10)	−0.0165 (7)
O2W	0.0263 (8)	0.0363 (8)	0.0347 (8)	−0.0067 (6)	0.0159 (7)	0.0062 (7)
O3	0.0255 (7)	0.0237 (7)	0.0278 (7)	0.0043 (6)	0.0133 (6)	−0.0037 (6)
O3W	0.0423 (9)	0.0299 (8)	0.0340 (8)	0.0112 (6)	0.0316 (7)	0.0079 (6)
O4	0.0431 (9)	0.0399 (9)	0.0229 (8)	0.0156 (8)	0.0050 (7)	−0.0029 (7)
O4W	0.0385 (9)	0.0376 (9)	0.0319 (8)	0.0052 (7)	0.0184 (7)	0.0049 (7)
O5	0.0211 (6)	0.0202 (7)	0.0193 (6)	0.0001 (5)	0.0143 (5)	−0.0006 (5)
C1	0.0207 (9)	0.0226 (10)	0.0260 (10)	0.0009 (8)	0.0171 (8)	−0.0017 (8)
C2	0.0252 (10)	0.0199 (9)	0.0236 (9)	−0.0014 (7)	0.0187 (8)	−0.0033 (7)
C3	0.0211 (9)	0.0180 (9)	0.0221 (9)	−0.0022 (7)	0.0143 (8)	−0.0024 (7)
C4	0.0207 (9)	0.0222 (9)	0.0246 (9)	−0.0016 (8)	0.0166 (8)	0.0004 (8)
C5	0.0328 (11)	0.0224 (10)	0.0317 (11)	−0.0002 (8)	0.0221 (10)	0.0059 (8)
C6	0.0273 (10)	0.0221 (10)	0.0304 (10)	0.0055 (8)	0.0168 (9)	0.0031 (8)
C7	0.0283 (10)	0.0266 (10)	0.0245 (10)	−0.0051 (8)	0.0200 (9)	−0.0036 (8)
C8	0.0235 (10)	0.0246 (10)	0.0220 (10)	0.0015 (8)	0.0144 (9)	0.0010 (8)

### Geometric parameters (Å, °)

**Table:**

Co1—O3	2.0631 (14)	O5—C1	1.459 (2)
Co1—O1W	2.0691 (15)	O5—C4	1.462 (2)
Co1—O2W	2.0728 (15)	C1—C6	1.526 (3)
Co1—O1	2.0849 (13)	C1—C2	1.542 (3)
Co1—O3W	2.0948 (13)	C1—H1A	0.9800
Co1—O5	2.1510 (13)	C2—C7	1.526 (3)
O1—C7	1.271 (2)	C2—C3	1.578 (2)
O1W—H1WA	0.8500	C2—H2A	0.9800
O1W—H1WB	0.8500	C3—C8	1.529 (3)
O2—C7	1.241 (2)	C3—C4	1.540 (3)
O2W—H2WA	0.8499	C3—H3A	0.9800
O2W—H2WB	0.8500	C4—C5	1.526 (3)
O3—C8	1.276 (2)	C4—H4A	0.9800
O3W—H3WA	0.8501	C5—C6	1.547 (3)
O3W—H3WB	0.8499	C5—H5A	0.9700
O4—C8	1.236 (2)	C5—H5B	0.9700
O4W—H4WA	0.8500	C6—H6A	0.9700
O4W—H4WB	0.8499	C6—H6B	0.9700
			
O3—Co1—O1W	93.29 (6)	C7—C2—C1	110.07 (15)
O3—Co1—O2W	173.18 (6)	C7—C2—C3	116.49 (15)
O1W—Co1—O2W	93.48 (6)	C1—C2—C3	101.13 (14)
O3—Co1—O1	85.86 (6)	C7—C2—H2A	109.6
O1W—Co1—O1	92.85 (6)	C1—C2—H2A	109.6
O2W—Co1—O1	92.92 (6)	C3—C2—H2A	109.6
O3—Co1—O3W	93.90 (6)	C8—C3—C4	110.57 (15)
O1W—Co1—O3W	90.60 (6)	C8—C3—C2	116.27 (15)
O2W—Co1—O3W	86.92 (6)	C4—C3—C2	101.03 (14)
O1—Co1—O3W	176.55 (5)	C8—C3—H3A	109.5
O3—Co1—O5	87.97 (5)	C4—C3—H3A	109.5
O1W—Co1—O5	176.73 (5)	C2—C3—H3A	109.5
O2W—Co1—O5	85.32 (5)	O5—C4—C5	102.24 (14)
O1—Co1—O5	90.25 (5)	O5—C4—C3	101.68 (13)
O3W—Co1—O5	86.30 (5)	C5—C4—C3	110.98 (15)
C7—O1—Co1	125.88 (12)	O5—C4—H4A	113.6
Co1—O1W—H1WA	129.4	C5—C4—H4A	113.6
Co1—O1W—H1WB	118.7	C3—C4—H4A	113.6
H1WA—O1W—H1WB	110.5	C4—C5—C6	101.94 (15)
Co1—O2W—H2WA	127.3	C4—C5—H5A	111.4
Co1—O2W—H2WB	118.7	C6—C5—H5A	111.4
H2WA—O2W—H2WB	109.9	C4—C5—H5B	111.4
C8—O3—Co1	122.32 (12)	C6—C5—H5B	111.4
Co1—O3W—H3WA	130.7	H5A—C5—H5B	109.2
Co1—O3W—H3WB	117.5	C1—C6—C5	101.65 (15)
H3WA—O3W—H3WB	102.8	C1—C6—H6A	111.4
H4WA—O4W—H4WB	105.2	C5—C6—H6A	111.4
C1—O5—C4	95.99 (13)	C1—C6—H6B	111.4
C1—O5—Co1	114.26 (10)	C5—C6—H6B	111.4
C4—O5—Co1	114.80 (10)	H6A—C6—H6B	109.3
O5—C1—C6	102.04 (14)	O2—C7—O1	122.60 (18)
O5—C1—C2	102.20 (14)	O2—C7—C2	118.71 (17)
C6—C1—C2	110.60 (16)	O1—C7—C2	118.60 (15)
O5—C1—H1A	113.6	O4—C8—O3	123.07 (18)
C6—C1—H1A	113.6	O4—C8—C3	119.02 (17)
C2—C1—H1A	113.6	O3—C8—C3	117.81 (16)
			
O3—Co1—O1—C7	−63.86 (16)	C1—C2—C3—C4	−1.55 (16)
O1W—Co1—O1—C7	−156.95 (17)	C1—O5—C4—C5	56.17 (15)
O2W—Co1—O1—C7	109.42 (16)	Co1—O5—C4—C5	176.41 (10)
O5—Co1—O1—C7	24.09 (16)	C1—O5—C4—C3	−58.60 (15)
O1W—Co1—O3—C8	140.00 (15)	Co1—O5—C4—C3	61.65 (14)
O1—Co1—O3—C8	47.38 (15)	C8—C3—C4—O5	−87.05 (16)
O3W—Co1—O3—C8	−129.17 (15)	C2—C3—C4—O5	36.63 (16)
O5—Co1—O3—C8	−43.02 (15)	C8—C3—C4—C5	164.82 (15)
O3—Co1—O5—C1	101.16 (11)	C2—C3—C4—C5	−71.49 (17)
O2W—Co1—O5—C1	−77.59 (12)	O5—C4—C5—C6	−33.84 (17)
O1—Co1—O5—C1	15.31 (12)	C3—C4—C5—C6	73.92 (18)
O3W—Co1—O5—C1	−164.80 (12)	O5—C1—C6—C5	35.70 (17)
O3—Co1—O5—C4	−8.38 (11)	C2—C1—C6—C5	−72.42 (18)
O2W—Co1—O5—C4	172.87 (11)	C4—C5—C6—C1	−1.04 (18)
O1—Co1—O5—C4	−94.23 (11)	Co1—O1—C7—O2	174.77 (16)
O3W—Co1—O5—C4	85.66 (11)	Co1—O1—C7—C2	−8.7 (3)
C4—O5—C1—C6	−56.90 (15)	C1—C2—C7—O2	126.16 (19)
Co1—O5—C1—C6	−177.57 (11)	C3—C2—C7—O2	−119.5 (2)
C4—O5—C1—C2	57.57 (15)	C1—C2—C7—O1	−50.5 (2)
Co1—O5—C1—C2	−63.10 (14)	C3—C2—C7—O1	63.9 (2)
O5—C1—C2—C7	89.68 (16)	Co1—O3—C8—O4	−151.18 (17)
C6—C1—C2—C7	−162.30 (15)	Co1—O3—C8—C3	32.4 (2)
O5—C1—C2—C3	−34.10 (16)	C4—C3—C8—O4	−140.06 (19)
C6—C1—C2—C3	73.92 (17)	C2—C3—C8—O4	105.6 (2)
C7—C2—C3—C8	−1.1 (2)	C4—C3—C8—O3	36.5 (2)
C1—C2—C3—C8	118.14 (16)	C2—C3—C8—O3	−77.9 (2)
C7—C2—C3—C4	−120.82 (16)		

### Hydrogen-bond geometry (Å, °)

**Table:**

*D*—H···*A*	*D*—H	H···*A*	*D*···*A*	*D*—H···*A*
O2W—H2WB···O4W	0.85	2.06	2.872 (2)	160.
O4W—H4WB···O5	0.85	2.60	3.0316 (19)	113.
O1W—H1WA···O4^i^	0.85	1.88	2.716 (2)	169.
O1W—H1WB···O4W^ii^	0.85	2.00	2.789 (2)	153.
O2W—H2WA···O1^iii^	0.85	1.87	2.7168 (19)	171.
O3W—H3WB···O2^iv^	0.85	1.84	2.688 (2)	173.
O4W—H4WB···O2^iv^	0.85	2.09	2.916 (2)	164.
O3W—H3WA···O3^v^	0.85	1.85	2.6969 (19)	178.
O4W—H4WA···O3W^vi^	0.85	2.35	3.112 (2)	149.

Symmetry codes: (i) −*x*+1, *y*+1/2, −*z*+1/2; (ii) −*x*+2, *y*+1/2, −*z*+3/2; (iii) −*x*+2, −*y*+2, −*z*+1; (iv) *x*, −*y*+3/2, *z*+1/2; (v) −*x*+1, −*y*+2, −*z*+1; (vi) −*x*+2, *y*−1/2, −*z*+3/2.
